# Protein Nitration in Patients with Mitochondrial Diseases

**DOI:** 10.3390/antiox14020211

**Published:** 2025-02-12

**Authors:** Jomênica B. Livramento, Gabriela S. Rodrigues, Jean Faber, Luis A. de Souza Filho, Felipo V. Moura, Camila D. S. Barros, Wladimir B. V. R. Pinto, Beny Schmidt, Acary S. B. Oliveira, Beatriz H. Kiyomoto, Célia H. Tengan

**Affiliations:** 1 Department of Neurology and Neurosurgery, Universidade Federal de São Paulo, São Paulo 04041-001, Brazil; jomenica@gmail.com (J.B.L.); gabis-rodrigues@hotmail.com (G.S.R.); jean.faber@unifesp.br (J.F.); l.darwin@hotmail.com (L.A.d.S.F.); felipovmoura@aol.com (F.V.M.); dantas.ca@hotmail.com (C.D.S.B.); wladimirbvrpinto@gmail.com (W.B.V.R.P.); acary.bulle@unifesp.br (A.S.B.O.); bhkiyomoto@unifesp.br (B.H.K.); 2 Department of Pathology, Universidade Federal de São Paulo, São Paulo 04041-001, Brazil; benyschmidt@gmail.com

**Keywords:** nitric oxide, mitochondrial diseases, protein nitration, oxidative stress, nitrative stress, mitochondrial DNA (mtDNA), reactive oxygen species (ROS), reactive nitrogen species (RNS)

## Abstract

Mitochondrial diseases are complex disorders caused by nuclear or mitochondrial DNA mutations, leading to oxidative phosphorylation deficiency and excessive production of reactive oxygen species (ROS). While ROS have been well established in the pathogenesis of these diseases, the role of reactive nitrogen species (RNS) remains unclear. In this study, we performed a quantitative analysis of muscle fibers to investigate the relationship between protein nitration and mitochondrial abnormalities (mitochondrial proliferation and cytochrome-c oxidase (COX) deficiency) and factors like genotype, muscle damage, and age. A total of 1961 muscle fibers (303 from 4 controls and 1658 from 29 patients with mitochondrial diseases) were analyzed by immunostaining for nitro-tyrosine. Contrary to previous findings, which identified nitro-tyrosine only in small muscle vessels, we observed a broader distribution affecting the sarcolemma and sarcoplasm. Using multivariate techniques, we identified a significant correlation between protein nitration and mitochondrial proliferation but found no associations with COX deficiency, age, muscle damage, or genotype. These findings suggest that nitrative stress may contribute to mitochondrial dysfunction or play a role in signaling processes that induce mitochondrial biogenesis. Our results provide new insights into the molecular mechanisms of mitochondrial diseases and highlight the potential relevance of protein nitration.

## 1. Introduction

Mitochondrial diseases are multisystem disorders characterized by genetic defects leading to oxidative phosphorylation (OXPHOS) deficiency [1]. They are caused by nuclear or mitochondrial DNA (mtDNA) mutations and are expressed with variable clinical manifestations, age of onset, and severity. Central nervous system (CNS) and skeletal muscle are frequently involved, but other manifestations are also reported, such as cardiomyopathy, visual impairment, and deafness [2]. Although genetic mutations have been reported increasingly due to next-generation sequencing, there is still no effective treatment for these diseases. The defective OXPHOS tends to increase the generation of reactive oxygen species (ROS) from the respiratory chain, making oxidative damage an additional pathogenic contributor to mitochondrial disease. Thus, common antioxidants are frequently prescribed despite no proven clinical effectiveness, while several new antioxidants have been tested in clinical trials [3].

In addition to ROS, reactive nitrogen species (RNS) can also be generated and predispose to nitrative stress. Tissues most affected by mitochondrial diseases, such as skeletal muscle and the brain, have an intracellular environment favorable to RNS generation due to the simultaneous presence of nitric oxide (NO) and ROS. In skeletal muscle, NO is produced by the two constitutive NO synthases (NOSs), neuronal and endothelial isoforms located close to ROS, generated in mitochondria. Excessive RNS production can lead to protein nitration, a reaction of NO2 radical and tyrosine residues in proteins, forming 3-nitro-tyrosine (3-NT). NO2 usually arises from the decomposition of peroxynitrite into NO2 and hydroxyl radicals (•OH), while peroxynitrite itself is formed through the reaction of NO and superoxide (O2-) [4,5]. Protein tyrosine nitration has been found in several diseases, including neurodegenerative diseases such as amyotrophic lateral sclerosis and Parkinson’s and Alzheimer’s diseases [6], and it was also reported to affect mitochondrial function [7]. Furthermore, the formation of 3-NT has been identified in proteins, such as dynamin-related protein 1 (Drp1), which is involved in the regulation of mitochondrial dynamics (fission and fusion), processes that modify mitochondrial morphology based on cellular energy and metabolic demands [7,8,9]. Dysregulation of mitochondrial dynamics can lead to neuronal degeneration and has been associated with the pathogenesis of several neurodegenerative diseases [10].

The formation of 3-NT can have positive and negative effects on protein function. In certain instances, tyrosine nitration can enhance protein activity and stability, as observed with the nitration of the catalytic subunit of protein kinase A (PKA) [11,12]. However, tyrosine nitration can also lead to protein dysfunction, contributing to disease development [7]. The causes and consequences of tyrosine nitration are complex and multifactorial, influenced by oxidative stress levels within the cell and the production of RNS such as NO and peroxynitrite [4]. Other key factors include NOS enzyme activity, antioxidant levels, and the availability of target proteins with tyrosine residues [4,5]. As the role of tyrosine nitration in disease pathogenesis becomes more recognized, understanding its mechanisms and consequences is crucial for developing new therapeutic strategies.

Skeletal muscle is a valuable tissue for studies on mitochondrial diseases due to the availability of diagnostic biopsies and histochemical reactions that can easily demonstrate mitochondrial abnormalities involving Complexes IV (cytochrome-c-oxidase, COX) and II (succinate dehydrogenase). Mitochondrial proliferation (ragged red fibers, RRFs) and COX deficiency are the most typical abnormalities in muscle histochemistry in patients with mitochondrial diseases. These abnormalities are usually found in a scattered pattern in mtDNA defects but are not obligatory for the diagnosis. Patients with genetic defects that do not affect Complex IV or II usually have a muscle biopsy without alterations. Skeletal muscle expresses the constitutive NOSs (endothelial and neuronal isoforms) with specific locations. The neuronal NOS is located in the sarcolemma, and the endothelial NOS is in the sarcoplasm [13], suggesting they may have different roles in the muscle cell. Alterations in NOS activity were detected in the skeletal muscle of patients with mitochondrial diseases and were localized in muscle fibers with mitochondrial abnormalities [14]. Increased NOS activity was observed in the sarcolemma of RRFs, suggesting that NO production is increased and predisposes to RNS generation [13,14].

In this study, we aimed to evaluate the presence of protein nitration in the skeletal muscle of patients with mitochondrial diseases with different genetic origins. We intended to verify whether protein nitration could be influenced by the type of mitochondrial abnormality (mitochondrial proliferation, COX deficiency, or both), genetic defect, muscle damage, and age.

## 2. Materials and Methods

### 2.1. Patients and Muscle Specimens

Muscle samples were from biopsies obtained exclusively for diagnostic purposes. This project was approved by the Research Ethics Committee at Universidade Federal de São Paulo. The study was performed with samples from 29 patients with mitochondrial diseases, defined by the presence of RRF, COX deficiency, or a pathogenic mutation in nuclear or mtDNA (Table 1). Genetic screening was performed by Southern blotting, Sanger sequencing of mtDNA, and a panel of nuclear genes related to mtDNA maintenance by next-generation sequencing.

Four muscle biopsy samples with no abnormalities or clinical evidence of metabolic or inflammatory disease served as controls. Samples were frozen in liquid nitrogen, stored at −80 °C, and submitted to serial cross-sections for histochemistry and immunostaining.

### 2.2. Histochemistry

Histochemical reactions were performed on 10 µm serial muscle sections for the enzymatic activity of succinate dehydrogenase (SDH, Complex II) and cytochrome c oxidase (COX, Complex IV) and incubated at 37 °C for 60 min [15]. For SDH histochemical reaction, the sections were incubated in 5 mM phosphate buffer, pH 7.6, containing 5 mM ethylene diamine tetraacetic acid (EDTA), 1 mM potassium cyanide, 0.2 mM phenazine methosulfate, 50 mM succinic acid, and 1.5 mM nitro blue tetrazolium. For COX staining, samples were incubated in 5 mM phosphate buffer, pH 7.4, containing 0.1% diaminobenzidine, 0.1% cytochrome c (from horse heart), and 0.02% catalase.

### 2.3. Immunostaining

Immunostaining was performed on 5 µm muscle sections, which were incubated for 10 min in acetone at 4 °C and blocked with 10% goat serum in phosphate buffered saline (PBS). After blocking, muscle samples were incubated with the primary antibody, polyclonal anti-nitrotyrosine (1:20 dilution, Invitrogen, Catalog # A21285, Waltham, MA, USA), overnight at 4 °C. Following this period, the sections were incubated for 1 h with the secondary antibody, anti-rabbit IgG Alexa FluorTM Plus 488 (1:200 dilution, Invitrogen, catalog # A32731). After washing with PBS, the coverslips were mounted on slides with 10% glycerol in PBS and were observed under a fluorescence microscope. 

**Figure 1:**
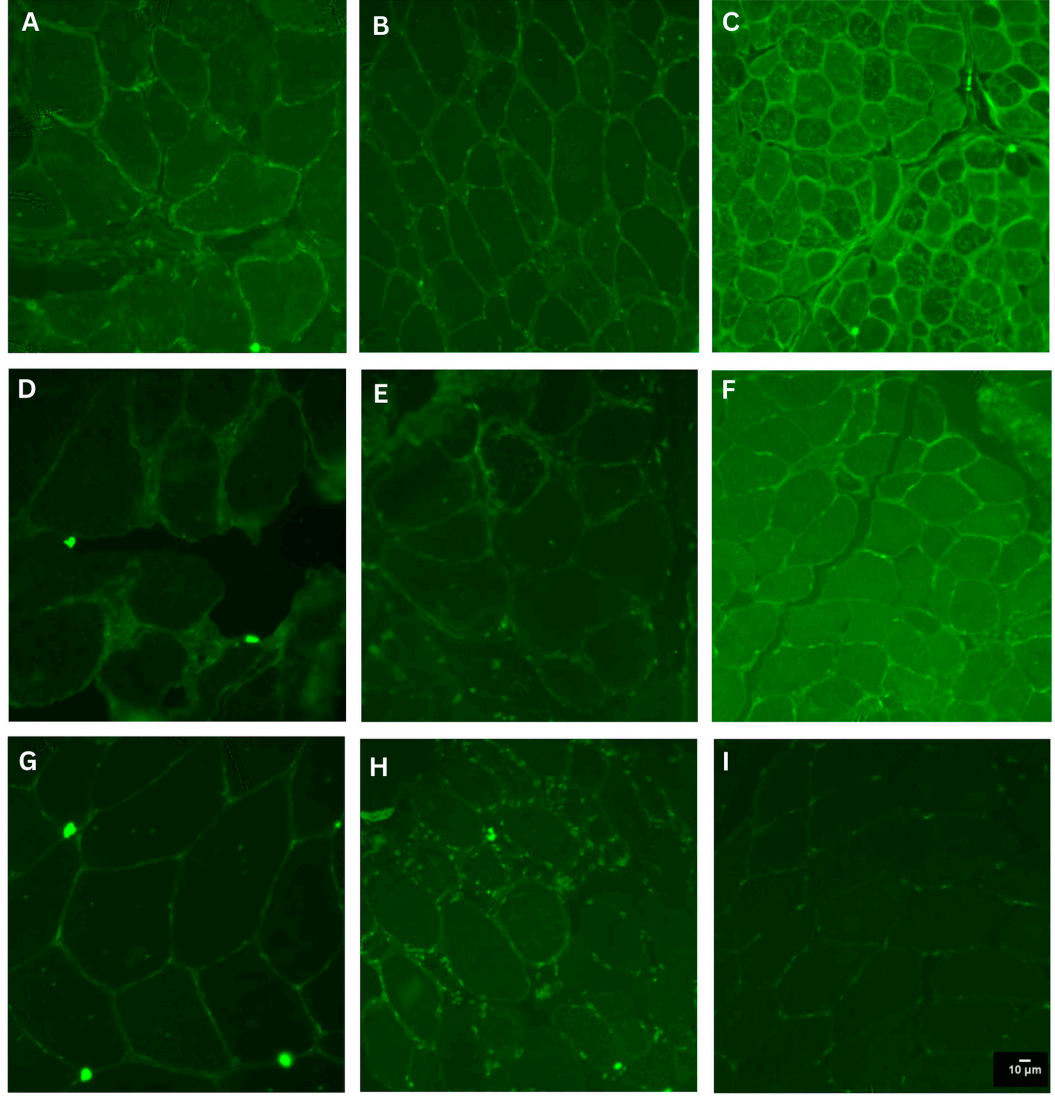
Detection of nitro-tyrosine (NT) by immunostaining. Representative images of NT-immunostaining: Panels (**A**) (P-19), (**B**) (P-20) and (**C**) (P-25) show patterns observed in muscle samples from patients with an *MT-TL1* mutation. Panels (**D**) (P-29) and (**E**) (P-6) show the pattern observed in patients with a single mtDNA deletion and an insertion in *MT-TQ* (P-22, (**F**)). Panels (**G**) (P-13) and (**H**) (P-18) show the patterns obtained in patients with nuclear gene mutations (*TK2*, not identified). Panel (**I**) shows a control sample (P-4). Scale bar shown in (**I**), is the same for (**A**–**H**).

We used a muscle biopsy specimen from a patient with polymyositis as a positive control, as nitrotyrosine immunoreactivity was previously reported in muscle fibers from these patients [16]. After testing several primary antibody concentrations, we used the dilution that resulted in the expected immunostaining in the positive control (Figure S1) and the minimum reactivity in the control samples (Figure 1I).

### 2.4. Immunostaining Quantification

Immunoreactivity images were captured with an Olympus digital camera with a 20X objective and saved with a TIFF extension. Positive fluorescence signals were quantified using the software ImageJ (version 1.52, NIH, USA). After splitting the image channels, the measurements were done on the green channel.

To assess the sarcolemmal nitro-tyrosine immunoreactivity (SL-NT), we measured several points distributed along the sarcolemma using the Multiple Points tool (dot-type point, tiny size). The location of each point was chosen with the aid of a 100 pixels^2^ grid on the image to ensure a non-biased selection (Figure S2A). The selected points were located where the horizontal or vertical line crossed the sarcolemmal membrane throughout the entire fiber perimeter. We measured the mean gray value of each selected point. Sarcoplasmic nitro-tyrosine immunoreactivity (SP-NT) was assessed by delimitating the area of the sarcoplasm of each fiber with the polygon selection line tool and measuring the mean optical intensity of the delimitated area (Figure S2B). A correction was made to ensure that measurements could be used to compare different slides. Each value was corrected by the background mean measured at four different points, and the results were expressed as a ratio (obtained value divided by background).

We considered a positive point when the value was above the highest value obtained in controls. SL-NT in each fiber was expressed as the percentage of positive points at the sarcolemmal membrane. For qualitative discrimination, we considered that the fiber had a positive SL-NT when the percentage of positive points was higher than zero.

### 2.5. Statistical Analysis

Parametric and non-parametric tests were applied considering the results of Shapiro–Wilk and Kolmogorov–Smirnov tests according to the Gaussian adherence of samples. Principal component analysis (PCA) was used to jointly evaluate the quantitative variables SL-NT, SP-NT, RRF, COXneg, and age. Creatine kinase (CK) levels were not included in the PCA due to missing values. The principal components (PCs) were retained when their associated eigenvalues were above one since they are more informative. Cluster discrimination was performed using the K-means algorithm with the Euclidean distance and silhouette technique to choose the best quantity of clusters (set in 2) [17].

For the genotype analysis, we classified the patients in groups considering the genetic etiology and the number of individuals with the same affected gene. The groups were identified as follows: (a) MT-TL1 (patients with mutations in *MT-TL1*, N = 6); (b) POLG (patients with mutations in *POLG*, N = 6); (c) single del (patients with a single large-scale deletion in mtDNA, N = 7); (d) other mtDNA genes (mutations in mtDNA with only one patient each, *MT-ND6*, *MT-TQ1*); (e) TK2 (patients with mutations in *TK2*, N = 2); (f) isolated nuclear genes (nuclear genes with only one patient each: *DGUOK*, *SLC25A4*, *MFN2*); (g) non-identified (non-identified nuclear gene defect, including one patient with multiple mtDNA deletions); and (h) normal (patients with muscle biopsies without alterations and no clinical suspicion of mitochondrial disease: P1–P4).

Combined phenotypes were used to classify the patients into three groups: (1) chronic progressive external ophthalmoplegia (CPEO); (2) mitochondrial myopathy (mitochondrial myopathy, exercise intolerance, or mitochondrial neuro-gastrointestinal encephalopathy, MNGIE,-like); and (3) mitochondrial encephalopathy (mitochondrial encephalopathy, Leigh syndrome, dystonia, or mitochondrial myopathy, encephalopathy, lactic acidosis, and stroke-like episodes, MELAS); in addition to (4) control (patients with no clinical manifestations suggestive of mitochondrial diseases, P1–P4).

Comparisons between clusters were performed with the unpaired t-test (quantitative variables) using its PC’s projections. The dependence of categorical variables was evaluated through Fisher’s exact test. One-way ANOVA with Dunnett’s post hoc test was used to compare results from different genotypes. Correlations were assessed through Pearson’s coefficient. Statistical significance was considered when *p* < 0.05. Graphs show mean and standard errors when a parametric test was applied, and median and 95% confidence interval with non-parametric tests. Statistical analyses were performed using GraphPad Prism 10 for MacOS (Version 10.2.3), Jamovi (Version 2.4.1.0), and MATLAB^®^ (Version 9.2.0 R2018a).

## 3. Results

Protein nitration was detected in the sarcolemma and sarcoplasm with variable degrees among the muscle fibers from patients and controls. In control muscle samples, immunoreactivity was low throughout the fibers, while in patients’ samples, the intensity of reactivity varied among different fibers and patients (Figure 1). The quantification of immunostaining intensity was performed in a total of 1961 muscle fibers (303 in 4 controls and 1658 in 29 patients), ranging from 52 to 125 fibers (mean: 57.17; SD: 33.60) in controls and from 19 to 139 fibers (mean: 54.75; SD: 25.02) in patients.

**Figure 2:**
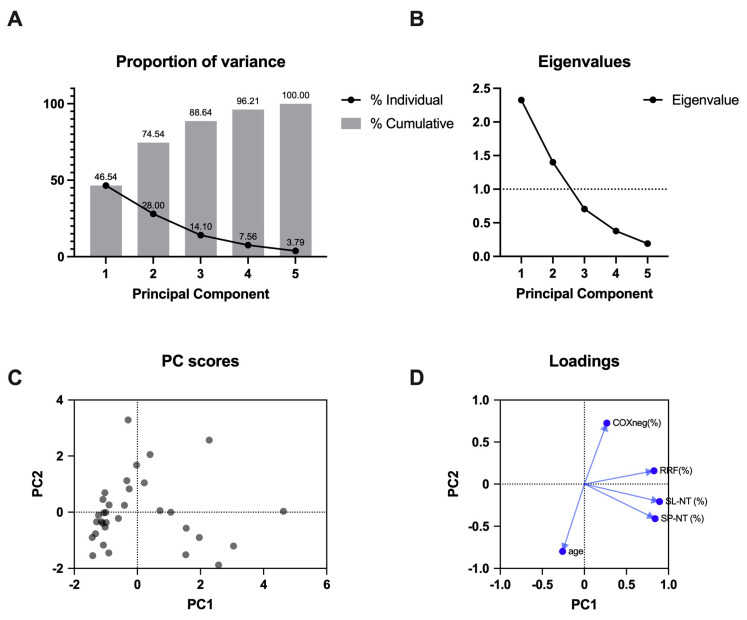
Principal component analysis (PCA). PCA revealed a cumulative proportion of variance of 74.54% with the first two PCs (principal components) and individual proportions of 46.54% with PC1 and 28% with PC2 considering the following variables: SL-NT, SP-NT, RRF, COXneg, and age (**A**). Eigenvalues are shown in (**B**), with only PC1 and PC2 values above 1. PC scores with PC1 vs. PC2 are shown in (**C**), and the biplot (**D**) demonstrates the loadings and directions for each variable.

We used a multiple variable approach (PCA) to identify closely related variables and find which features could be related to nitro-tyrosine immunoreactivity (Spreadsheet S1). We analyzed the following variables: age, SL-NT (percentage of positive fibers), SP-NT (percentage of positive fibers), mitochondrial proliferation (percentage of RRFs), and COX deficiency (percentage of COXneg fibers). PCA reduced these five variables into two dimensions, principal components 1 (PC1) and 2 (PC2), which together explained 74.54% of the data variance (Figure 2A,B). SL-NT, SP-NT, and RRF contributed with high loadings to PC1, while COXneg and age contributed to PC2, but in opposite directions (Table 2, Figure 2D). SL-NT and SP-NT were strongly correlated with a 0.79 correlation coefficient (Table 3). The proportion of RRF was also correlated with SL-NT and SP-NT with moderate positive coefficient correlations (0.57 and 0.54, respectively), suggesting that the proportion of RRF is partially related to the increment in SL-NT and SP-NT. The correlation coefficients among the variables (RRF, SL-NT, SP-NT, COXneg, and age) suggest that only RRF is related to nitro-tyrosine immunoreactivity (Table 3).

**Figure 3:**
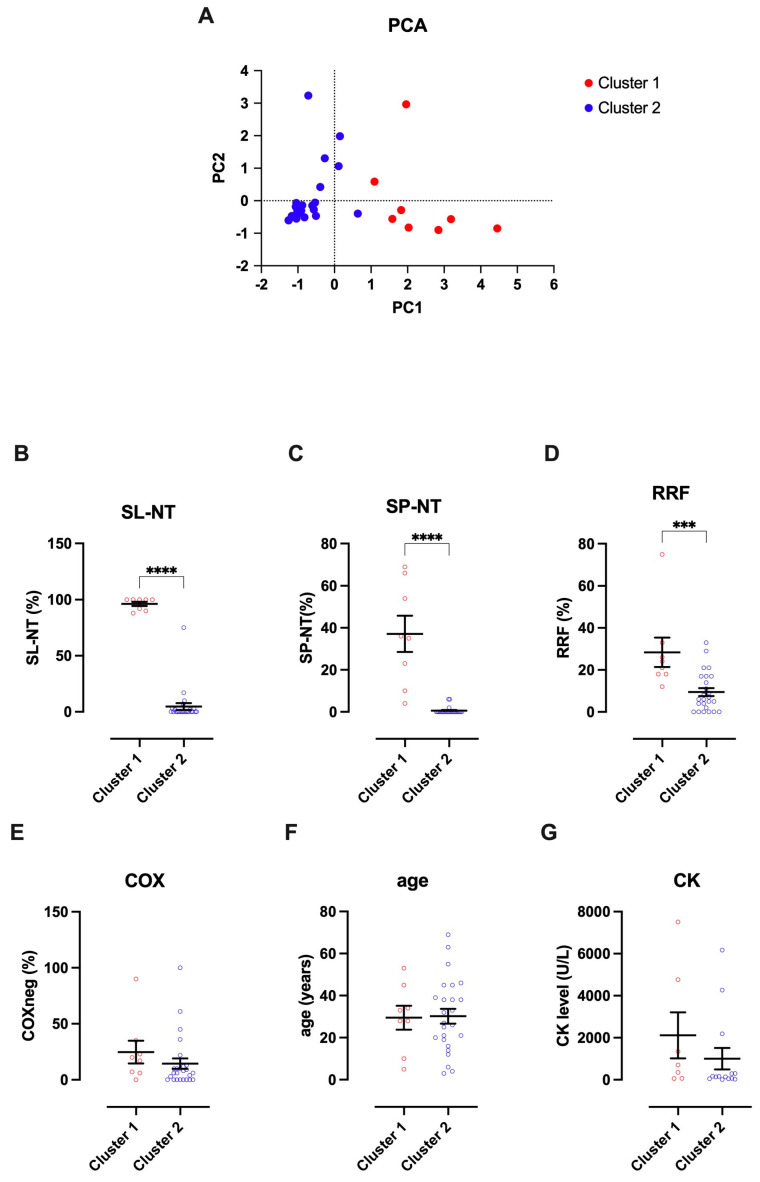
Comparisons between Clusters 1 and 2. The K-means algorithm identified two clusters with Euclidean distance (**A**). Samples aggregated in Cluster 1 demonstrate a higher percentage of SL-NT+ (**B**), SP-NT+ (**C**), and RRF (**D**). The groups did not differ when considering the proportion of COXneg fibers (**E**), age (**F**), and CK level (**G**); unpaired t-test, *** *p*= 0.0008; **** *p* < 0.0001.

A cluster analysis on the PC1 vs. PC2 plot showed that the scores could be discriminated in two clusters, named Cluster 1 and Cluster 2 (Figure 3A). Cluster 1 had higher proportions of SL-NT, SP-NT, and RRFs (Figure 3B–D). Comparisons between Clusters 1 and 2, considering the proportion of COXneg, age, and CK level (not included in the PCA due to missing values), did not show any differences between the clusters, suggesting that these features did not impact NT immunostaining in our group of samples (Figure 3D).

We also evaluated whether a specific genotype or phenotype was more predominant in one of the clusters. Due to statistical constraints, the genotype analysis included only the groups with more than five patients (*POLG*, *MT-TL1*, and single del). Phenotype analysis used groups with combined phenotypes, as described in Section 2.5 (CPEO, mitochondrial myopathy, and mitochondrial encephalopathy. The analysis did not reveal any predominant genotype (Table 4) or phenotype (Table 5) in any cluster.

**Figure 4:**
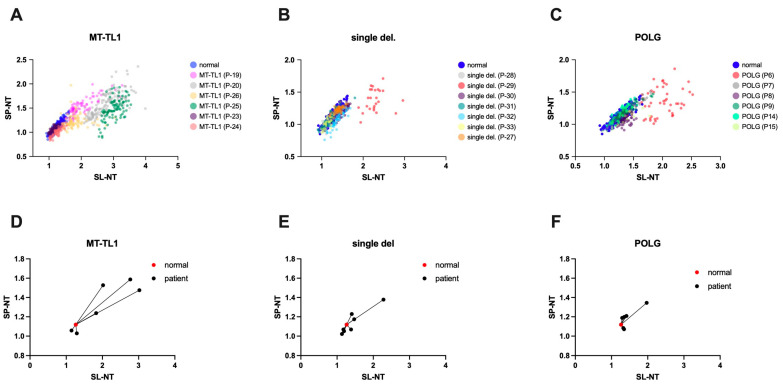
SL-NT and SP-NT immunoreactivity intensities in muscle fibers of patients with *MT-TL1* and *POLG* mutations and single mtDNA deletions (single del). The figure shows the distribution of SL-NT and SP-NT intensities in individual muscle fibers from patients with *MT-TL1* mutations (**A**), *POLG* mutations (**C**), and single mtDNA deletions (**B**). The scatterplots show that some patients exhibit distributions distinct from the control group. These differences are highlighted in panels (**D**–**F**), demonstrating the distances between each patient’s centroid and the normal centroid. The *MT-TL1* group shows a pattern of greater distances than the other groups (single mtDNA deletions and *POLG* mutations). However, no statistically significant differences were found among the groups (one-way ANOVA, *p* = 0.0691).

To further explore the relationship between genotype and nitro-tyrosine immunostaining, we analyzed the intensities of nitro-tyrosine immunostaining in individual fibers of each patient (Spreadsheet S2). Clusters were constructed on graphs with the intensities of variables SL-NT and SP-NT for each patient. In this way, we could compare the genotype groups (*MT-TL1*, *POLG*, and single del) to the normal group (Figure 4). The graphs revealed that SL-NT was the most significantly altered variable across patient groups. The group *MT-TL1* exhibited the most dispersed clusters, with four patients’ clusters separating from the normal group. *POLG* and single del clusters were more concentrated around the normal group, with only one patient separated from the normal cluster in each group.

**Figure 5:**
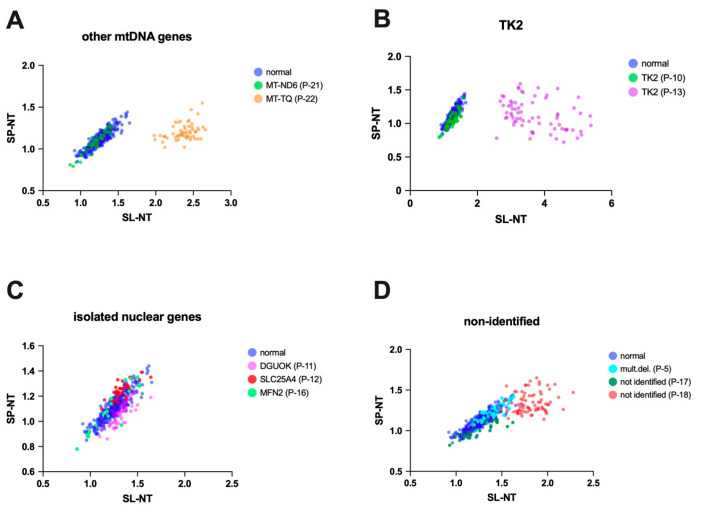
SL-NT and SP-NT immunoreactivity intensities in muscle fiber isolated cases with various genotypes. The figure shows the distribution of SL-NT and SP-NT intensities in individual muscle fibers in the groups: other mtDNA genes (**A**), TK2 (**B**), isolated nuclear genes (**C**), and non-identified (**D**). Blue dots represent the normal group. The scatterplots demonstrate cases where data points deviate from the control group (P-22 in (**A**), P-13 in (**B**), P-18 in (**D**)).

In order to have measurable data for statistical comparison, we quantified the distance between each patient and the control cluster using the centroid distances (mean SL-NT and SP-NT values; Spreadsheet S2; Figure 4D–F). Although the *MT-TL1* group showed the largest centroid distances, no genotype group displayed larger distances from the control cluster (*MT-TL1*: 0.84 ± 0.29; single del: 0.28 ± 0.13; *POLG*: 0.21 ± 0.11; one-way ANOVA, *p* = 0.07). Some patients also showed distinct separation from controls in the other genotype groups (other mtDNA genes, TK2, isolated nuclear genes, and non-identified; Figure 5). However, the small number of patients in each group does not allow statistical analysis. Interestingly, in the TK2 group, even though the two patients had a mutation in the same gene, only one was separated from the normal cluster, primarily due to elevated SL-NT. These findings suggest that factors beyond genotype may contribute to the heterogeneous distribution of SL-NT levels.

**Figure 6:**
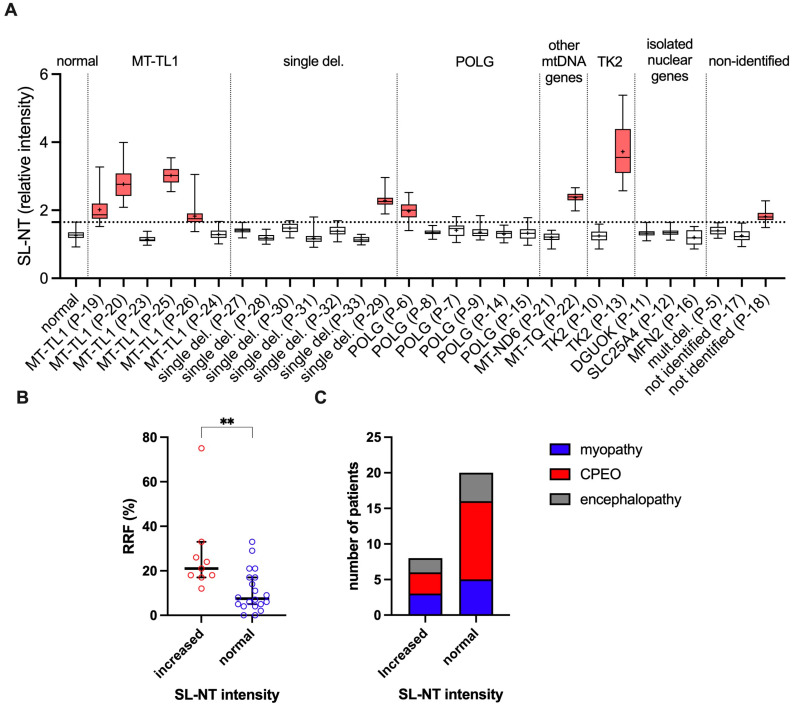
Comparison of RRF proportions and phenotype distribution based on SL-NT immunoreactivity intensity. (**A**) SL-NT intensities of each patient are displayed. We considered samples with increased SL-NT intensity (red box plots) when the mean intensity exceeded the upper limit of the normal samples (dotted line). (**B**) The proportion of RRF is significantly higher in patients with increased SL-NT compared to those with normal SL-NT intensities (medians and 95% confidence intervals are represented; Mann–Whitney test; ** *p* = 0.0021). (**C**) The distribution of clinical phenotypes (myopathy, CPEO, and encephalopathy) remains similar between groups with increased and normal SL-NT intensity, indicating no significant variation in phenotype distribution. CPEO: chronic progressive external ophthalmoplegia; RRF: ragged red fiber; SL-NT: sarcolemmal nitro-tyrosine. Box plot margins represent the 25th and 75th percentiles, the horizontal line is the median, + is the mean, and whiskers are the minimum and maximum values.

Most patients’ clusters deviated from the normal group due to increased SL-NT immunoreactivity. Thus, we classified the patients according to the SL-NT immunoreactivity intensity, considering “increased SL-NT” those values exceeding the upper limit of the normal group (Figure 6A). We then compared the RRF proportions and phenotype distribution between patients with “increased SL-NT” and those with “normal SL-NT”. This comparison showed higher proportions of RRF associated with increased SL-NT (Figure 6B), reinforcing the link between SL-NT levels and RRF, as identified in the PCA analysis. There was no difference in the phenotype distribution (Figure 6C).

It is interesting to note that four out of six patients with *MT-TL1* mutation presented increased SL-NT. In contrast, in other groups, such as those with mutations in *POLG* or mtDNA single deletion, increased SL-NT was found only in a minority of patients, i.e., one out of six in *POLG* and one out of seven in mtDNA single deletion. However, the small sample size does not allow a conclusion regarding the propensity of increased SL-NT immunoreactivity in samples with MT-TL1 mutation.

## 4. Discussion

Despite advances in understanding the pathogenesis of mitochondrial diseases, the role of protein nitration still needs to be explored. In skeletal muscle, the expression of constitutive NOS isoforms [13], along with ROS generated by a dysfunctional electron transport chain, creates a favorable environment for nitrative stress and protein modifications, such as nitration. This post-translational modification can alter protein function, leading to either a loss or gain of function, depending on the affected protein [18,19]. Furthermore, nitration can interfere with tyrosine-based modifications like phosphorylation, disrupting critical cell signaling pathways [18].

Nitration of essential proteins for mitochondrial processes, including those within the electron transport chain and the tricarboxylic acid cycle, has previously been shown to impair mitochondrial function and disrupt cellular energy homeostasis [7,20]. A previous study identified nitro-tyrosine exclusively in small blood vessels within the skeletal muscle of patients with mitochondrial diseases, suggesting the contribution of nitro-oxidative stress in stroke-like episodes by reducing NO bioavailability [21]. Our study revealed the presence of nitro-tyrosine in other regions of the muscle, suggesting a more widespread role for nitro-oxidative stress in mitochondrial pathology. The differences in immunostaining patterns between these studies may be attributed to variability in antibody specificity. Peptide-based immunoprecipitation experiments demonstrated that only 373 out of 977 nitrotyrosine-containing proteins were commonly detected by four antibodies in a myeloma cell line treated with peroxynitrite [22]. One, two or three antibodies detected the remaining peptides [22].

Mitochondrial diseases are often associated with disruptions in the antioxidant defense; thus, an imbalance in antioxidant defenses could increase susceptibility to nitrative stress [23]. This hypothesis is supported by a proteomic analysis in two patients with the same *TWNK* mutation. In this study, the more symptomatic patient had downregulation in several antioxidant defense proteins and a higher proportion of RRFs [23]. This downregulation of antioxidant enzymes is consistent with the existing literature showing that protein nitration can impair antioxidant defenses by inactivating key antioxidant enzymes, such as manganese superoxide dismutase (Mn-SOD), through nitration at the critical Tyr34 residue [24,25]. In addition, Mn-SOD nitration has been observed in the small muscle vessels of patients with mitochondrial diseases, further supporting the role of nitrative stress in disease pathogenesis [21]. Notably, these small muscle vessels also exhibit increased mitochondrial content, which supports our multivariate analysis, revealing a significant correlation between protein nitration and mitochondrial proliferation, observed as RRF in the skeletal muscle of patients with mitochondrial diseases. However, due to the limited size of the muscle specimens, we did not have enough material to identify the nitrated proteins through a proteomic analysis.

RRF is regarded as a compensatory response to cellular energy deficits, suggesting that mitochondrial proliferation may result from mitochondrial deficiency [26,27]. Therefore, the correlation between protein nitration and RRF raises the possibility that protein nitration could play a role in the cellular response to mitochondrial dysfunction or contribute to mitochondrial deficiency. However, we did not find any correlation between protein nitration and mitochondrial dysfunction, which we evaluated in this study as COX deficiency. This lack of association is likely because COX deficiency represents only one aspect of mitochondrial dysfunction. Mitochondrial diseases can also involve deficiencies in other respiratory chain complexes, such as Complex I, III and V, which are not detectable through standard muscle histochemistry [28].

Beyond its association with mitochondrial deficiency, tyrosine nitration has also been linked to various cellular signaling pathways, including platelet activation, yeast mating events, and rat embryos’ heart development [29]. However, the exact biological relevance of these findings remains unclear [29]. Another possibility in the case of mitochondrial pathways is that protein nitration may be involved in signaling processes that regulate or induce mitochondrial proliferation. This hypothesis is supported by studies demonstrating that NO induces mitochondrial biogenesis in muscle cells [30] and low levels of peroxynitrite in MnSOD-knocked-down NRK cells [31].

While our study provides valuable insights into the role of protein nitration in mitochondrial diseases, certain factors should be considered when interpreting the findings. First, the small sample size limited our ability to draw firm conclusions regarding the influence of different genotypes, phenotypes, and muscle damage. The rarity of mitochondrial diseases makes it difficult to include a larger number of patients for each genotype, which would allow a better assessment of genotype-specific patterns of protein nitration. In our sample group, only three genotypes had a sufficient size (six or seven patients) for statistical analysis: patients with mutations in *POLG* and *MT-TL1* and those with a single large-scale mtDNA deletion. Unfortunately, other genotypes were underrepresented, with only one or two patients per group, and in two cases, the genetic defect was unidentified. Even with this small cohort, we observed a trend toward increased nitrated proteins in the MT-TL1 group, though the difference was not statistically significant. A larger group could confirm this difference.

Second, we were constrained by the limited availability of control samples. Since muscle biopsy specimens are typically obtained for diagnostic purposes, most patients had suspected muscle abnormalities. Although a larger control group could strengthen the statistical power of our findings, the current study still offers valuable insights.

Third, although the specificity of the NT-immunoreactivity is supported by the co-localization of NOS isoforms in skeletal muscle, as previously reported [13], we cannot fully exclude the possibility of nonspecific staining. To minimize the effect of nonspecific immunostaining, we considered only signals above the normal control threshold as positive staining.

Finally, our evaluation of mitochondrial dysfunction was limited to COX deficiency, which captures only one aspect of OXPHOS dysfunction. Mitochondrial diseases can involve deficiencies in other electron transport chain complexes (Complex I, III, and V) that are not detectable through standard muscle histochemistry.

## 5. Conclusions

In summary, our findings suggest a potential role for protein nitration in the pathogenesis of mitochondrial diseases, particularly through its effects on mitochondrial function and antioxidant defenses. The correlation between nitrate proteins and mitochondrial proliferation suggests that nitrative stress may contribute to mitochondrial dysfunction or be part of the signaling process that induces mitochondrial biogenesis. However, further studies are still necessary to elucidate the precise mechanisms by which protein nitration influences mitochondrial disease pathology and to explore potential therapeutic strategies targeting nitrative stress.

## Figures and Tables

**Table 1:** Characteristics of selected patients.

Patient ID	Mitochondrial Syndrome	Genetic Defect		Age	Gender	CK (U/L)	RRF (%)	COXneg (%)
		mtDNA	Nuclear Gene					
P-1	Ctrl (not applicable)	Not applicable		12	M			
P-2	Ctrl (not applicable)	Not applicable		6	M			
P-3	Ctrl (not applicable)	Not applicable		32	F			
P-4	Ctrl (not applicable)	Not applicable		46	F			
P-5	CPEO	Multiple deletions	Not identified	69	M	-	5	9
P-6	CPEO	Multiple deletions	*POLG*; p.Gly848Ser (heteroz.)	53	F	58	18	20
P-7	CPEO	Multiple deletions	*POLG*; p.Tyr955Cys (heteroz.)	63	F	298	6	10
P-8	MNGIE-like	Multiple deletions	*POLG*; p.Ala889Thr (heteroz.)	33	M	-	4	13
P-9	MNGIE-like	Multiple deletions	*POLG*; p.Ala889Thr (heteroz.)	38	F	-	14	12
P-10	CPEO	Multiple deletions	*TK2*; p.Lys202del (homoz.)	21	F	158	21	22
P-11	Mitochondrial myopathy	Multiple deletions	*DGUOK*; p.Gln170Arg (heteroz)	45	F	-	9	10
P-12	CPEO	Multiple deletions	*SLC25A4;* D93_K96insDKY (heteroz.)	45	F	167	2	3
P-13	Mitochondrial myopathy	No rearrangements	*TK2*; p.Thr108Met (homoz.)	33	M	1346	12	35
P-14	Mitochondrial myopathy	No rearrangements	*POLG*; p.Glu1143Gly (heteroz.)	21	M	4270	7	4
P-15	Mitochondrial myopathy	No rearrangements	*POLG*; p.His613Tyr (heteroz.)	20	M	6170	17	0
P-16	CPEO	No rearrangements	*MFN2;* p.Arg468His (heteroz.)	25	M	2188	6	0
P-17	Leigh Syndrome	-	Not identified	3	M	-	0	100
P-18	Mitochondrial myopathy	No rearrangements	Not identified	28	F	7510	24	7
P-19	Exercise intolerance	*MT-TL1*; m.3243A>G (56%)		45	M	700	18	17
P-20	CPEO	*MT-TL1*; m.3251A>G (55%)		34	F	355	26	23
P-21	Dystonia	*MT-ND6*; m.14459G>A (100%)		26	M	nl	0	0
P-22	CPEO	*MT-TQ*; m.4370_4371insT (87%)		10	M	-	33	90
P-23	MELAS	*MT-TL1*; m.3243A>G (46%)		4	M	12	17	8
P-24	Dystonia	*MT-TL1*; m.3243A>G (60%)		55	F	nl	11	0
P-25	Mitochondrial encephalopathy	*MT-TL1*; m.3243A>G (56%)		5	M	64	75	0
P-26	MELAS	*MT-TL1*; m.3243A>G (67%)		19	M	-	17	6
P-27	CPEO	Single deletion (99%)		14	F	47	21	45
P-28	CPEO	Single deletion (68%)		27	F	132	33	36
P-29	CPEO	Single deletion (65%)		28	M	4762	21	6
P-30	CPEO	Single deletion (69%)		16	M	69	29	61
P-31	CPEO	Single deletion (60%)		38	F	28	8	6
P-32	CPEO	Single deletion (30%)		38	M	139	5	6
P-33	CPEO	Single deletion (60%)		39	M	53	4	10

Ctrl: control; CPEO: Chronic progressive external ophthalmoplegia; MNGIE: Mitochondrial neuro-gastrointestinal encephalopathy; MELAS: Mitochondrial myopathy, encephalopathy, lactic acidosis, and stroke-like episodes; F: female; M: male; heteroz.: heterozygous; homoz.: homozygous; RRF: ragged red fiber; COXneg: cytochorme c oxidase negative fiber; mtDNA: mitochondrial DNA; CK: creatine kinase; ID: identification; -: data not available. The percentage of mutant mtDNA is shown between parentheses after the mtDNA defect.

**Table 2:** Principal component analysis (PCA) loadings.

Variable	PC1	PC2
SL-NT (%)	0.89	−0.21
SP-NT (%)	0.84	−0.41
RRF (%)	0.83	0.16
COXneg (%)	0.27	0.73
Age	−0.26	−0.80

SL-NT: sarcolemmal nitro-tyrosine immunoreactivity, SP-NT: sarcoplasmic nitro-tyrosine immunoreactivity, PC1: principal component 1, PC2: principal component 2.

**Table 3:** Correlation matrix between variables.

	SL-NT (%)	SP-NT (%)	RRF (%)	COXneg (%)	Age
SL-NT (%)	1	0.79	0.57	0.16	−0.04
SP-NT (%)	0.79	1	0.54	−0.04	0.07
RRF (%)	0.57	0.54	1	0.19	−0.33
COXneg (%)	0.16	−0.04	0.19	1	−0.34
Age	−0.04	0.07	−0.33	−0.34	1

**Table 4:** Distribution of samples classified by genotype in the different clusters.

	Clusters		
Genotype	Cluster 1	Cluster 2	Total
Normal	0 (0%)	4 (12.1%)	4 (12.1%)
POLG	1 (3.0%)	4 (12.1%)	5 (15.2%)
MT-TL1	3 (9.1%)	3 (9.1%)	6 (18.2%)
Single del	1 (3.0%)	6 (18.2%)	7 (21.2%)
Total	8 (24.2%)	25 (75.8%)	22 (100.0%)

The two-sided Fisher’s exact test showed no significant association between genotype and Cluster 1 or 2; (%): percentages of the total.

**Table 5:** Distribution of samples classified by phenotype in the different clusters.

	Clusters		
Phenotype	Cluster 1	Cluster 2	Total
Control	0 (0.0%)	4 (12.1%)	4 (12.1%)
CPEO	4 (12.1%)	11 (33.3%)	15 (45.5%)
Mitochondrial myopathy	3 (9.1%)	5 (15.2%)	8 (24.2%)
Mitochondrial encephalopathy	1 (3.0%)	5 (15.2%)	6 (18.2%)
Total	8 (24.2%)	25 (75.8%)	33 (100.0%)

The two-sided Fisher’s exact test showed no significant association between phenotype and Cluster 1 or 2; (%): percentages of the total.

## Data Availability

The original contributions presented in this study are included in the article/Supplementary Material. Further inquiries can be directed to the corresponding author (C.H.T.).

## Supplementary Materials

The following supporting information can be downloaded at: https://www.mdpi.com/article/10.3390/antiox14020211/s1, Figure S1: Positive control for nitrotyrosine immunostaining. Examples of positive staining in muscle fibers are shown with arrows. Figure S2: The image shows the print screen on Image J with the grids used to select the points to quantify the sarcolemmal immunostaining (A) and the sarcoplasm area selection (B); Spreadsheet S1: PCA data; Spreadsheet S2: NT intensity in individual fibers.
