# Supplementary material for: Protein Nitration in Patients with Mitochondrial Diseases
Source: Antioxidants (Basel). 2025 Feb 12;14(2):211. doi: 10.3390/antiox14020211 (PMC11852069; doi:10.3390/antiox14020211)
Supplement: Supplementary file 1 [file antioxidants-14-00211-s001.zip › Supplementary Files captions.pdf]

Supplementary Files captions

Figure S1. Positive control for nitrotyrosine-immunostaining.
Nitro-tyrosine immunostaining in muscle sample from a patient with polymyositis. The arrows show examples of positive staining in the sarcolemma of muscle fibers.

Figure S2: Image quantification method.
The image shows the print screen on Image J with the grids used to select the points to quantify the sarcolemmal immunostaining (A) and the sarcoplasm area selection (B);

Spreadsheet S1: PCA data

Spreadsheet S2: NT intensity in individual fibers.
