# Supplementary figures and images for: Protein Nitration in Patients with Mitochondrial Diseases
Source: Antioxidants (Basel). 2025 Feb 12;14(2):211. doi: 10.3390/antiox14020211 (PMC11852069; doi:10.3390/antiox14020211)

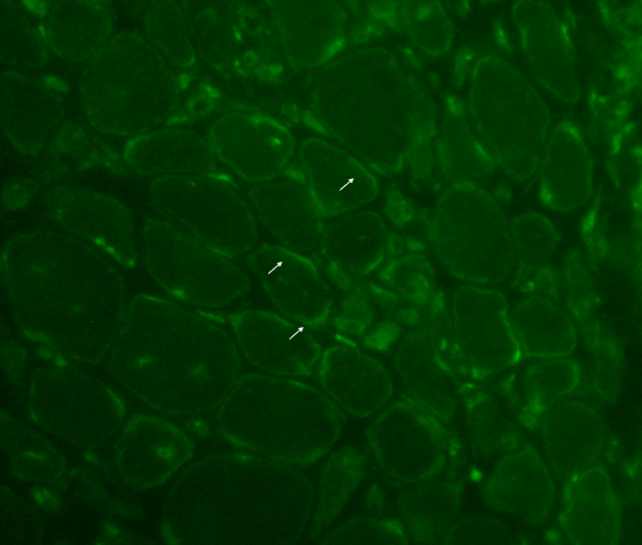

Supplement: Supplementary file 1 [file antioxidants-14-00211-s001.zip › Figure S-1.tiff]

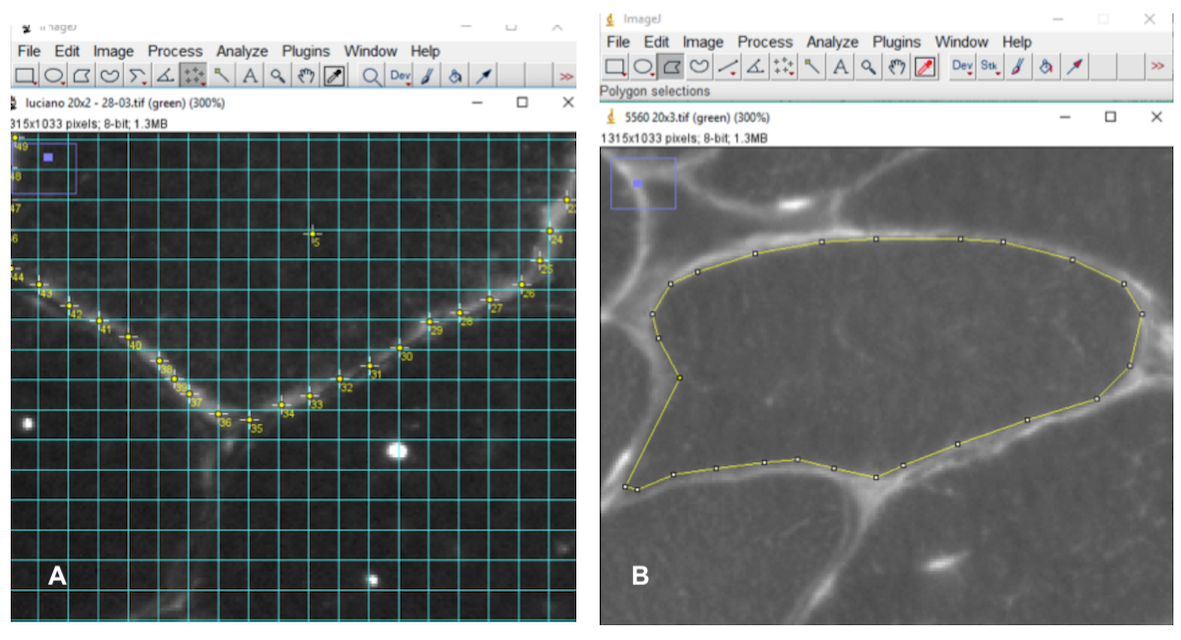

Supplement: Supplementary file 1 [file antioxidants-14-00211-s001.zip › Figure S-2.png]
